# Label-Free Proteomic Analysis of Flavohemoglobin Deleted Strain of* Saccharomyces cerevisiae*

**DOI:** 10.1155/2016/8302423

**Published:** 2016-01-11

**Authors:** Chiranjit Panja, Rakesh K. S. Setty, Gopal Vaidyanathan, Sanjay Ghosh

**Affiliations:** ^1^Department of Biochemistry, University of Calcutta, 35, Ballygunge Circular Road, Kolkata, West Bengal 700 019, India; ^2^Waters India Pvt. Ltd., Bangalore 560 058, India

## Abstract

Yeast flavohemoglobin, YHb, encoded by the nuclear gene YHB1, has been implicated in the nitrosative stress responses in* Saccharomyces cerevisiae*. It is still unclear how* S. cerevisiae* can withstand this NO level in the absence of flavohemoglobin. To better understand the physiological function of flavohemoglobin in yeast, in the present study a label-free differential proteomics study has been carried out in wild-type and YHB1 deleted strains of* S. cerevisiae* grown under fermentative conditions. From the analysis, 417 proteins in Y190 and 392 proteins in ΔYHB1 were identified with high confidence. Interestingly, among the differentially expressed identified proteins, 40 proteins were found to be downregulated whereas 41 were found to be upregulated in ΔYHB1 strain of* S. cerevisiae* (*p* value < 0.05). The differentially expressed proteins were also classified according to gene ontology (GO) terms. The most enriched and significant GO terms included nitrogen compound biosynthesis, amino acid biosynthesis, translational regulation, and protein folding. Interactions of differentially expressed proteins were generated using Search Tool for the Retrieval of Interacting Genes (STRING) database. This is the first report which offers a more complete view of the proteome changes in* S. cerevisiae* in the absence of flavohemoglobin.

## 1. Introduction

All major groups of organisms including plants, vertebrates, invertebrates, protozoa, bacteria, and fungi contain hemoglobin [1]. Hemoglobin constitutes a diverse superfamily of proteins that are grouped together because they all bind oxygen reversibly and possess a conserved heme-binding domain, the “myoglobin fold” [2]. Beside these common features, hemoglobin is otherwise divergent in structure and complexity. Most types of vertebrate hemoglobin consist of two types of polypeptide subunits that have single heme domains and form tetrameric oligomers. However, bacterial, fungal, and protozoan hemoglobin fall into two general types: (i) dimeric hemoproteins composed of two single heme domain polypeptides and (ii) monomeric flavohemoproteins containing a single heme-binding domain at the amino terminus and a carboxyl-terminal FAD-binding domain that is related to those found in proteins in the ferredoxin-NADP reductase family [3]. DNA sequence analysis showed that this flavohemoglobin appears to form a distinct subgroup within the hemoglobin family [1, 4]. It has been known for a long time that vertebrate hemoglobin and invertebrate hemoglobin transport O_2_ and CO_2_. Now it has been discovered that the hemoglobin of both eukaryotes and prokaryotes has additional functions relating to nitric oxide [NO]. Studies showed that expression of the* E. coli* hemoglobin gene (hmp) is induced by nitrite, NO, and SNO and confer protection from each of them.* E. coli* mutants deficient in HMP show compromised ability to metabolize NO and SNO to nitrate [5–9]. Prominent roles of flavohemoglobin protein (HMP) have been demonstrated in NO metabolism and* Salmonella* pathogenesis [10, 11]. Flavohemoglobin mutant* S. typhimurium* shows impaired growth under nitrosative stress conditions [12].

*Saccharomyces* hemoglobin was discovered in 1953 [13] but its function remained unknown for a long time [4, 14, 15]. The expression of its gene, YHB1, is induced by oxygen, which is the opposite of the effect oxygen has on the expression of most bacterial hemoglobin genes [16, 17]. This observation has been taken to indicate that the hemoglobin of yeast and bacteria may have different functions. It has been shown that yeast flavohemoglobin (yhb1) localizes to two distinct intracellular compartments in respiring cells, the mitochondrial matrix and the cytosol [18]. Moreover, it has been found that the distribution of YHB1 between these two compartments is affected by the presence or absence of oxygen and by the mitochondrial genome. Several observations suggest physiological connections between the expression and function of this protein, mitochondrial respiration, and oxidative and nitrosative stress. It has now been shown that yeast YHB1 is required to metabolize NO and thereby protects against nitrosylation of cellular targets and inhibition of cell growth under both aerobic and anaerobic conditions. That is, the primary function of YHB1 is to protect against a nitrosative stress. It has also been suggested that YHB1 may protect against oxidative stress [19], but this function has been questioned [20]. Our laboratory for the first time investigated a detailed characterization on the effect of nitrosative stress on* S. cerevisiae* under respiratory proficient condition. The lack of flavohemoglobin was apparently not detrimental to the cell. Our studies showed that NO or reactive nitrogen species (RNS) produced in flavohemoglobin mutant (ΔYHB1) strain along with the wild-type strain (Y190) of* S. cerevisiae* under respiratory proficient conditions. Neither the respiration rate nor the ROS production level was changed in ΔYHB1 strains of* S. cerevisiae* compared to wild type indicating the existence of second line of defense in absence of flavohemoglobin. In this context it is interesting to note that increased basal levels of GSH, GR, and mitochondrial catalase appear to be able to compensate for the lower levels of cellular flavohemoglobin [21]. We observed many immunopositive spots both in cytosol and in mitochondria from Y190 and ΔYHB1 using monoclonal anti-3-nitrotyrosine antibody indicating a basal level of NO or nitrite or peroxynitrite is produced in yeast system [22, 23]. To identify proteins nitrated in vivo we analyzed mitochondrial proteins from Y190 strains of* S. cerevisiae*. Among the eight identified proteins, two target mitochondrial proteins are aconitase and isocitrate dehydrogenase that are involved directly in the citric acid cycle. This investigation is the first comprehensive study to identify mitochondrial proteins nitrated in vivo [22]. In another study, we also showed that induction of catalase was found at activity as well as gene expression level in ΔYHB1 mutants when subtoxic dose of peroxynitrite was applied to wild type as well as in ΔYHB1 mutant grown under fermentative conditions. Such induction was not due to intracellular reactive oxygen species formation [24].

It is still unclear whether endogenously produced NO, if not scavenged by flavohemoglobin, has any detrimental role or how yeast can withstand this NO level. To better understand the physiological function of flavohemoglobin in yeast, in the present study we have carried out label-free differential proteomics study using soluble extract of wild-type (Y190) and flavohemoglobin deleted strains (ΔYHB1 mutant) of* S. cerevisiae* grown under fermentative conditions. Quantification was achieved by employing a label-free system, whereby the summed intensity of the top three most intense peptides assigned to a protein is assumed to be proportional to the protein concentration, which can therefore be estimated by comparison with the three largest peptide intensities of an injected internal standard. This study could provide a significant insight into the role of flavohemoglobin on metabolic pathways or how* S. cerevisiae* cope with and regulate in vivo produced NO and RNS.

## 2. Materials and Method

### 2.1. Strains and Media Used

Strains of* Saccharomyces cerevisiae* used in the studies were Y190 (*MATa GAL 4 GAL 80 HIS3-Δ200 TRP1-901 ADE2-101 URA3-52 LEU2-3,112 URA3::GAL1-LacZ LYS2::GAL4 (UAS)::HIS3 cyh* ^R^, wild type) from CLONTECH and its isogenic flavohemoglobin (YHB1) null mutant (both the strains were gifted by Dr. Jonathan Stamler) [4]. The cells were grown in YPD broth (1% yeast extract (Difco), 2% Bacto-Peptone (Difco), and 2% dextrose) at 30°C under shaking condition (140–150 rpm).

### 2.2. Measurement of Cell Growth

For growth measurement experiments, midlog phase culture was used as inoculum following dilution in fresh media to O.D._600_ nm = 0.07–0.10. Cell growth was monitored turbidimetrically by measuring the absorbance at 600 nm at every one-hour interval.

### 2.3. Cell Cultivation and Cell Lysate Preparation

Single colony of wild-type* S. cerevisiae* (Y190) and flavohemoglobin deleted strain of* S. cerevisiae* (ΔYHB1) were grown in YPD medium under shaking condition at 150 rpm overnight at 30°C. A very small amount of overnight grown inoculum of both the strains was inoculated in fresh YPD media so that the initial O.D. of the culture was 0.1. The inoculated cultures were grown under shaking condition at 150 rpm for 12 hours. Both the cultures reached at 15 O.D. which was the midlog phase of* S. cerevisiae* growth. Midlog phase cells of Y190 and ΔYHB1 of* S. cerevisiae* were collected following centrifugation at 5000 rpm for 5 minutes. Cells were washed twice in PBS followed by washing in double distilled water and were kept on ice. Temperature was maintained at 4°C throughout the lysate preparation. Yeast cells were lysed using glass bead lysis method. Cells were suspended in 10 mM Tris-HCl with protease inhibitor cocktail (Sigma). Acid washed glass beads were added and were subjected to vortex for 1 min followed by immediately keeping the lysate in ice for 1 min. This cycle was followed at least six times until sufficient numbers of cells were found to be lysed. Protein concentrations were measured using Bradford assay [25]. Samples were lyophilized and stored at −20°C for further LC/MS analysis.

### 2.4. Sample Preparation for LC

An amount of 200 *μ*g of each protein sample was solubilized in 0.1% Rapigest (w/v Rapigest in 50 mM ammonium bicarbonate). The solution was concentrated to 100 *μ*L using 3 kDa spin column and heated to 80°C for 30 min. Samples were reduced with 0.5 mM DTT at 60°C for 30 min followed by alkylated with 5 *μ*L of 200 mM iodoacetamide for 30 min in dark at room temperature. All the samples were digested with trypsin, where trypsin to protein ratio was maintained at 1 : 50 for 4-hour incubation at 37°C and with additional incubation with trypsin at 1 : 50 ratio overnight at 37°C. The sample protein digests were acidified to hydrolyze Rapigest and to stop trypsin activity with 2 *μ*L formic acid (37% w/v). Tryptic digest was mixed with standard protein digest and used for nano-UPLC/ESI QToF HDMS^E^ analysis using SYNAPT G2 HDMS.

### 2.5. LC-MS^E^

For nano-HPLC analysis, a NanoACQUITY UPLC/ESI QToF HDMS^E^ with SYNAPT G2 HDMS system configured for conventional 1D chromatography was used. Analytical reversed-phase column used was NanoACQUITY C18, 1.7 *μ*m, 75 *μ*m × 200 mm, ethylene bridged hybrid (BEH), Waters. For peptide trapping, a trapping column NanoACQUITY UPLC column, Symmetry C18 5 *μ*m, 180 *μ*m × 20 mm, Waters, was used. Mobile solvents were 0.1% formic acid in water as buffer A and 0.1% formic acid in acetonitrile as buffer B. Injection volume was set at 1 *μ*L. Peptides were eluted from the column in a 60 min linear gradient going from 99% buffer A to 1% buffer B in 60 min. The flow rate during elution was set at 300 nL/min and the analytical column temperature was set at 37°C. All samples were analyzed in triplicate. For all measurements, the mass spectrometer was operated in the positive-ion mode with a typical resolving power of at least 10,000 full-width half-maximum. Accurate mass LC-MS data were collected in high definition MS^E^ mode at low energy using low energy of 4 eV and for elevated energy ramping from 15 to 40 eV, switching every 0.8 sec. The total acquisition time was 60 min for the mass range of 50–2000 *m*/*z*. The lock mass and window were set at 785.8426/0.25 Da. Raw data was imported to Waters PLGS software and processed using low energy threshold of 100 counts and higher energy threshold of 30 counts. Intensity threshold was set at 500 counts.

### 2.6. Data Processing, Protein Identification, and Quantification

LC-MS data were processed and searched using ProteinLynx Global Server version 2.3 (PLGS 2.3) (Waters). Raw datasets were processed and peak lists were generated based on the assignment of precursor ions and fragments based on similar retention times as described previously [26, 27].* S. cerevisiae* UNIPROT databank was used to search each triplicate run with the following parameters: peptide tolerance was 10 ppm and 20 ppm for fragment tolerance; trypsin missed cleavages were set as 1 and the following fixed modification was considered: CAM, Acetyl N-term, oxidation (M), deamidation (NQ), and nitric oxide NO (CY). Label-free quantitation was performed using peak intensity measurements in Waters ExpressionE, which is part of PLGS 2.3. Quantitation is done assuming the intensity response under ESI conditions of the three most intense peptides intensities observed in low collision energy mode in a triplicate set is a function of the molar amount infused in the mass spectrometer. For protein quantification, datasets were normalized using the PLGS “autonormalization” function and clustering software included in PLGS 2.3. The absolute amount of every identified protein is determined as a ratio of the “Hi3” peptide intensity of the protein of interest to that of the “Hi3” peptide intensity of a spiked internal standard as reference. Only those proteins identified in at least two of three injections were used as significant change in protein abundance. All proteins whose abundance was significantly different between samples were manually assessed by checking the matched peptide and replication level across samples which may be due to highly similar protein isoforms.

### 2.7. Statistical Analysis

For each quantification, the *p* value was calculated for the log_2_ transformed values by using independent sample *t*-test. The *p* value was calculated based on the normal distribution of the ratios. Proteins with a *p* value below 0.05 were considered statistically significant.

### 2.8. Functional Annotation

The significantly regulated proteins were analyzed using Database for Annotation, Visualization, and Integrated Discovery (DAVID, version 6.7) functional annotation tool [28, 29]. Functionally enriched gene ontology (GO) terms were visualized in semantic space using SimRel functional similarity measure [30] and the REViGO online visualization tool [31] modified with the Cytoscape version 3.1.1. Protein interaction networks were built using the online database resource Search Tool for the Retrieval of Interacting Genes (STRING) [32] which are visualized by Medusa [33], a Java application for visualizing and manipulating graphs of interaction. The interactions include direct (physical) and indirect (functional) associations derived from genomic context, high-throughput experiments, coexpression, and literature mining.

## 3. Results

Many studies have shown that yeast flavohemoglobin encoded by the gene YHB1 consumes NO very efficiently and Yhb1 is likely to be the main protective mechanism against NO in some microorganisms [4, 16, 34, 35]. To investigate the role of flavohemoglobin in* S. cerevisiae* under fermentative growth, label-free quantitative differential proteomic profiling was carried out using cell lysates of Y190 and ΔYHB1 strain of* S. cerevisiae.* Differential proteomic profiling in ΔYHB1 strain of* S. cerevisiae* would also reflect the change in expression pattern of the nitrosative stress responsive proteins.

**Figure 1:**
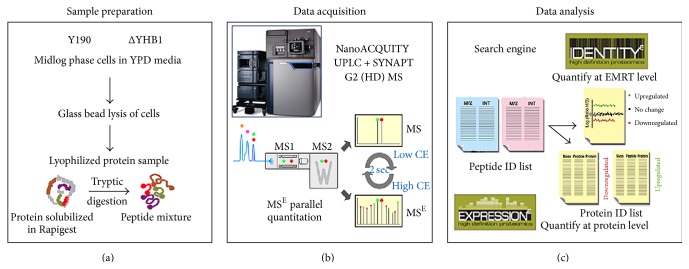
Overall workflow for label-free quantitation of Y190 and ΔYHB1 strains of* S. cerevisiae* using differential proteomics. Midlog phase cells were lysed and protein extracts were digested with trypsin (a), followed by LC-MS/MS runs in MS^E^ parallel mode (b) and statistical evaluation of results for identification and quantitation of identified proteins (c).

**Figure 2:**
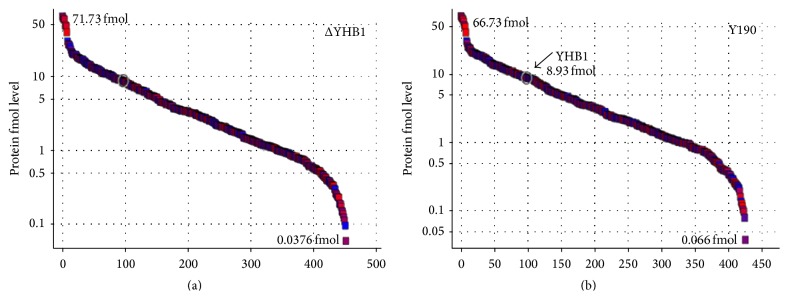
Scattered plot of the dynamic range of proteins identified in ΔYHB1 (a) and Y190 (b) strains of* S. cerevisiae*. *x*-axis represents number of proteins identified in label-free quantitation which not only includes proteins identified from the data base of* S. cerevisiae* but also includes the other homologues of it. *y*-axis represents proteins at fmol level. The arrow indicates the level of flavohemoglobin protein (8.93 fmol) in wild-type (Y190) strain of* S. cerevisiae* which is absent in flavohemoglobin deleted strain of* S. cerevisiae* (ΔYHB1).

**Figure 3:**
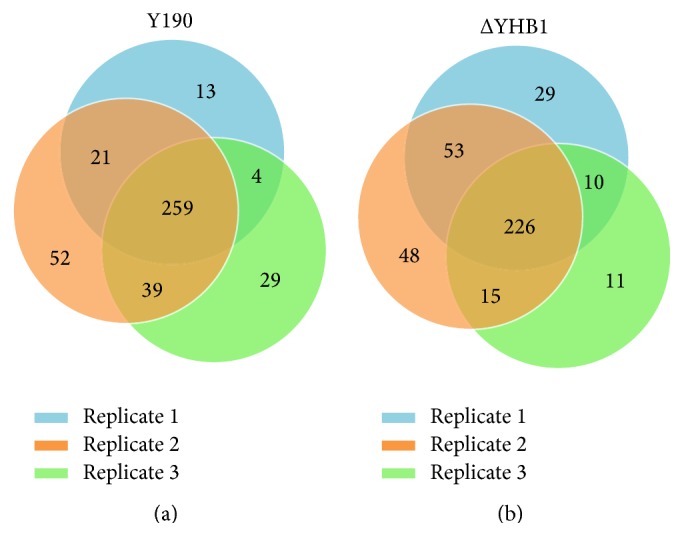
Venn diagrams for proteome comparison in three technical replicate of Y190 (a) and ΔYHB1 (b) strains of* S. cerevisiae*. Three color codes represent individual replicate of label-free proteome of Y190 and ΔYHB1 of* S. cerevisiae*. The intersections of the Venn diagram include proteins which have been identified in replicative experiments.

The quantitative differential proteomics experiments (Figure 1) were carried out in Waters NanoACQUITY UPLC coupled to a SYNAPT G2 mass spectrometer.  Figure 2 shows a plot of abundance relating to 498 proteins in Y190 (Figure 2(a)) and 473 proteins in ΔYHB1 strain of* S. cerevisiae* (Figure 2(b)), respectively, identified from YPD grown cell extracts. A total list of identified proteins with protein score, sequence coverage, top three matched peptide intensities, and calculated protein quantity in fmol and nanogram levels in Y190 and ΔYHB1 strain of* S. cerevisiae* are shown in Supplementary Table S1 and Supplementary Table S2 in Supplementary Material available online at http://dx.doi.org/10.1155/2016/8302423, respectively. However, the above numbers for identified proteins also include the other homologues of* S. cerevisiae*. Therefore, the identified proteins were analyzed based on the availability in* S. cerevisiae* data base exclusively.  Figure 3 represents the complete differential proteomic profiling of Y190 and ΔYHB1 strain of* S. cerevisiae*. The protein identification was based on the detection of at least two fragment ions per peptide with two or more than two peptides identified per protein. During the merging of the individual datasets from the label-free experiment, only peptides with a False Discovery Rate (FDR) < 4% were included. From the analysis, 417 proteins in Y190 and 392 proteins in ΔYHB1 were identified with high confidence. Among the total proteins identified in wild-type Y190, 259 proteins were identified in triplicate and 64 proteins in duplicate while 94 proteins were identified in single set of experiment (Figure 3(a)). On the other hand, in ΔYHB1 strain of* S. cerevisiae*, 227 proteins were identified in triplicate and 78 proteins in duplicate while 88 proteins were identified in single set of experiment (Figure 3(b)). Based on the fold change values, upregulated and downregulated proteins were arranged with their corresponding *p* value (Supplementary Table S3). Only those proteins were considered as positive up- or downregulated proteins which were identified with a *p* value < 0.05 and were found to become abundant in at least two of the three replicates. Among the 81 nonredundant differentially expressed identified proteins, 40 were found to be downregulated (Table 1) whereas 41 were found to be upregulated (Table 2) in ΔYHB1 strain of* S. cerevisiae.* Nine proteins were identified as unique proteins exclusively present in Y190 strain of* S. cerevisiae* with high confidence in triplicate results (Table 3). It is important to note that flavohemoglobin was present with an abundance of 8.33 fmol in average of the three replicates in the list of nine unique proteins present in Y190 strain of* S. cerevisiae*. However, only 4 proteins were identified as unique which were exclusively present in ΔYHB1 strain of* S. cerevisiae* in triplicate (Table 3).

**Figure 4:**
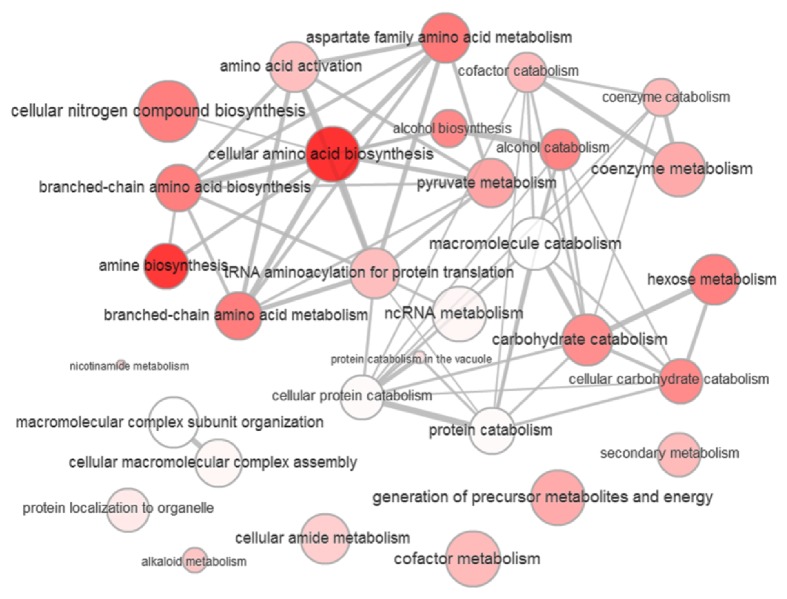
Enriched gene ontology terms among the upregulated and downregulated proteins upon flavohemoglobin YHB1 deletion in* S. cerevisiae*. The intensity of color and diameter of each dot represent the enrichment of the GO terms.

**Figure 5:**
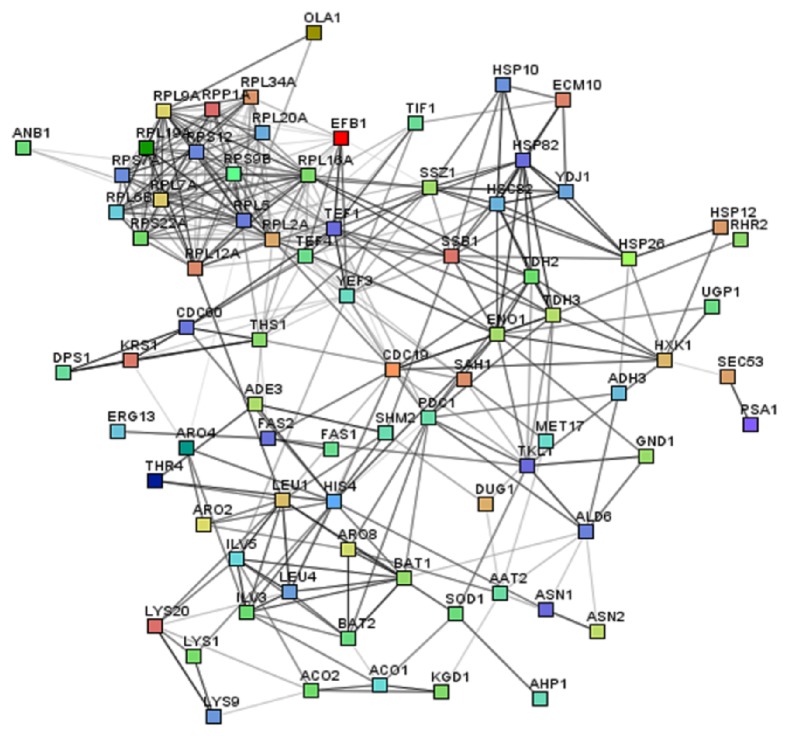
Differentially expressed proteins of ΔYHB1 strain of* S. cerevisiae* compared with wild-type Y190 as depicted in their interaction networks by STRING and visualized by Medusa. Each node represents the upregulated and downregulated proteins of ΔYHB1 strain of* S. cerevisiae*. The edges represent putative protein interactions recorded or predicted by STRING.

To gain insight into the proteome level change that occurred from YHB1 deletion, the whole proteomics data (*p* value < 0.05) were subjected to different functional analysis tools. First, the data was analyzed using the Database for Annotation, Visualization, and Integrated Discovery (DAVID) v6.7 functional annotation tool. DAVID uses gene ontology and other data sources to cluster proteins based on the shared annotations to similarity cluster. These annotation clusters help to visualize the connections shared by different proteins in various categories within gene ontology and other annotation sources. Sixty-three of the regulated proteins (both up- and downregulated) were clustered into 14 clusters with similarity term overlap and threshold for kappa similarity set at 4 and 0.35, respectively. Group membership was kept at 2 and multiple lineage threshold was set at 0.5 (Supplementary Table S4). The differentially expressed proteins were also classified according to gene ontology (GO) terms. Sixty-four of the enriched GO terms were visualized using SimRel functional similarity measure and REViGO online visualization tool modified with CYTOSCAPE v3.1.1. The most enriched and significant GO terms were the following pathways: nitrogen compound biosynthesis, amino acid biosynthesis, translational regulation, and protein folding (Figure 4, Supplementary Table S5). Interactions between the regulated set of proteins were generated using Search Tool for the Retrieval of Interacting Genes (STRING) database. When the seventy-nine of the regulated proteins were mapped it was found that the regulated proteins were significantly interacting with each other (Figure 5, Supplementary Table S6).

Flavohemoglobin deletion in* S. cerevisiae* caused significant impact on amino acid biosynthetic pathways. The most affected amino acid biosynthetic processes include the synthesis of aspartate family of amino acids, branched chain amino acids, and aromatic amino acids where downregulation was observed for 19 proteins involved in the biosynthesis of amino acids. Histidine biosynthesis trifunctional protein His4p was found to be maximally downregulated in ΔYHB1 strain of* S. cerevisiae* protein (log_2_-fold change −2.82, *p* value: 0.014). It was the highest fold change value obtained in quantitative label-free differential proteomic profiling.

Another important protein was Arg1p which is required for arginine biosynthesis. Arg1p was uniquely identified in wild-type* S. cerevisiae* Y190 in this experiment. Two of the proteins responsible for antioxidant defense Peroxiredoxin Ahp1p and superoxide dismutase Sod1p were also found to be downregulated in ΔYHB1 strain of* S. cerevisiae*. Other major downregulated proteins include three of the amino acyl tRNA synthases Dps1, Krs1, and Ths1, serine hydroxymethyltransferase Shm2p, mitochondrial alcohol dehydrogenase Adh3p, and Cys Gly metallopeptidase Dug1p.

Many members of the glycolytic, citrate cycle (TCA cycle), and pentose phosphate pathway were also affected upon YHB1 deletion. Enolase (Eno1p) of glycolytic pathway, aconitase (Aco1p and Aco2p), and 2-oxoglutarate dehydrogenase (Kgd1p) of the TCA cycle were all downregulated in ΔYHB1. Some other members of the TCA cycle phosphoenolpyruvate carboxykinase (Pck1p), NAD (+)-dependent isocitrate dehydrogenase (Idh1p), and pyruvate carboxylase cytoplasmic isoform (Pyc1p) were also uniquely identified in Y190. However, two isoforms of glyceraldehyde-3-phosphate dehydrogenase (Tdh2p and Tdh3p), pyruvate kinase Cdc19p, pyruvate decarboxylase Pdc1p, and hexokinase Hxk1p were upregulated in ΔYHB1. Also, E1 beta subunit of the pyruvate dehydrogenase (PDH) complex Pdb1p was uniquely identified in ΔYHB1. 6-Phosphogluconate dehydrogenase Gnd1p and transketolase Tkl1p of the pentose phosphate pathway were also upregulated in ΔYHB1 strain of* S. cerevisiae*.

Few of the chaperone proteins were differentially regulated upon YHB1 deletion. Hsp90 chaperone Hsc82p and the Hsp40/DnaJ family chaperone Ydj1p were upregulated in ΔYHB1 strain of* S. cerevisiae*. However, other Hsp70 family members Ssb1p and Ecm10p and the other Hsp90 chaperone Hsp82p were found to be downregulated in ΔYHB1 strain of* S. cerevisiae*. Other cytosolic members of small heat shock family proteins Hsp10p, Hsp12p, and Hsp26p were also downregulated in ΔYHB1 strain of* S. cerevisiae*.

Few members of the amino sugar and nucleotide sugar metabolism were found to be upregulated in ΔYHB1 strain of* S. cerevisiae*. These included mannose-1-phosphate guanylyltransferase (Psa1p), phosphomannomutase (Sec53p), and UTP-glucose-1-phosphate uridylyltransferase (Ugp1p). Two of the fatty acid synthase subunits Fas1p and Fas2p, hydroxymethylglutaryl-CoA synthase Erg13p, and Sah1p which regulates cellular lipid homoeostasis were upregulated in ΔYHB1 strain of* S. cerevisiae*. Clathrin heavy chain Chc1p, GTP-binding protein Ola1p, few of the ribosomal proteins of the 60S and 40S, DEA(D/H)-box RNA helicase Tif1p, and few of the elongation factors Yef3p, Tef4p, Efb1p, and Tef1p were also upregulated ΔYHB1 strain of* S. cerevisiae*.

## 4. Discussion

This is first proteomics based study to illustrate the cellular response in flavohemoglobin deleted strain of* S. cerevisiae*. Flavohemoglobin deletion in* S. cerevisiae* did not impose any significant change in the yeast phenotype or growth pattern between the wild type and mutant when cells were grown under fermentative conditions as evident from the growth curve data [23]. It proves that* S. cerevisiae* can cope with the reactive nitrogen species (RNS) generated from cellular metabolisms even when those RNS are not scavenged by flavohemoglobin. Moreover, our previous studies also indicated the microbiostatic effect of RNS on* S. cerevisiae* under nitrosative stress even in the absence of flavohemoglobin [21]. This indicates that other compensating and more elaborate mechanisms are present in yeast to meet this challenge.

A global transcriptome profile of* S. cerevisiae* under nitrosative stress has been reported by Horan et al., 2006 [35]. The transcriptomics data were collected from the cells which were grown in respiratory proficient media. Our proteomics study was carried out in fermentative YPD media and it did not match with the transcriptomics data. Much less work has been published on cellular defense against nitrosative stress; particularly the role of cellular metabolic regulation as defense against nitrosative stress is poorly understood. Our data shows that branched chain amino acids (BCAA) leucine, isoleucine, and valine biosynthesis were impaired in ΔYHB1 strain of* S. cerevisiae* due to downregulation of dihydroxy-acid dehydratase (Ilv3p) that catalyzes the second step of leucine biosynthesis. Its immediate downstream enzymes Ilv5p, aminotransferases Bat1p/Bat2p, and isopropylmalate isomerase Leu1p were also downregulated in ΔYHB1. Ilv3p is a vulnerable target to nitrosative stress as it contains Fe-S cluster that is prone to be attacked by nitric oxide [36].

The downregulation of amino acid biosynthesis I in ΔYHB1 strain of* S. cerevisiae* also affects lysine biosynthesis via the *α*-aminoadipate which is the dominant pathway of lysine biosynthesis in* S. cerevisiae* [37, 38]. Lys20p is the key enzyme in first step of lysine biosynthesis that converts *α*-ketoglutarate to homocitrate and exerts maximum flux control in lysine synthesis [39]. It was downregulated in flavohemoglobin deleted strain of* S. cerevisiae*. Other lysine biosynthesis enzymes that were also downregulated include saccharopine dehydrogenase Lys1p and Lys9p. Also, aminoadipate aminotransferase Aro8p that catalyses the formation of *α*-aminoadipate from *α*-ketoadipate [40] was found to be downregulated in ΔYHB1 strain of* S. cerevisiae*. Other pathways like asparagine biosynthesis (Asn1p and Asn2p), shikimate acid pathway and chorismate biosynthesis via shikimate (Aro4p, Aro8p, and Aro2p), and histidine biosynthesis (His3p) were downregulated in the absence of flavohemoglobin. However, the downregulation of the enzymes in amino acid biosynthesis process was not prominent in ΔYHB1 strain of* S. cerevisiae* in this study as cells were grown in amino acid rich complex media under fermentative conditions.

Similar impact on amino acid biosynthesis under nitrosative stress has already been reported in* E. coli*. When* E. coli* cells are challenged with nitric oxide, it induces bacteriostasis by damaging the Fe-S cluster of dihydroxy-acid dehydratase involved in branched chain amino acid synthesis [41]. Nitric oxide also induces lysine and methionine auxotrophy in* Salmonella typhimurium* that results from reduced succinyl-CoA availability that is required for methionine and lysine biosynthesis due to lipoamide dehydrogenase deactivation by nitric oxide [42].

Block in TCA cycle due to NO has been documented in many previous studies [43, 44]. In the present study, downregulation of aconitase (Aco1p) which is also a Fe-S cluster containing protein and 2-oxoglutarate dehydrogenase (Kgd1) was observed in ΔYHB1 strain of* S. cerevisiae*. The supply of 2-oxoglutarate exerts significant control on the lysine synthesis flux in* S. cerevisiae* [45]. Thus, substrate unavailability due to downregulation of TCA cycle may result in the downregulation of lysine biosynthesis as observed in ΔYHB1 strain of* S. cerevisiae*. Another direct effect of the downregulation in amino acid biosynthesis was observed in protein biosynthesis in which three of the amino acyl synthases were downregulated. But the effect may be compensated by upregulation of RNA helicases like Tif1p and elongation factor like Yef3p, along with some of the ribosomal proteins. Tif1p (eIF4A) and its homologs in higher eukaryotes were found to be essential for translation initiation [46–48].

Many of the heat shock proteins and chaperones which are required for effective protein folding were also regulated in absence of YHB1. Hsp70 chaperone Ssb1p and Hsp70 Ecm10p involved in mitochondrial protein import [49] were downregulated in ΔYHB1 strain of* S. cerevisiae*. Another protein Ola1p, a member of the Obg family of GTP-binding proteins, shown to specifically interact with HSP70 [50] was also upregulated in ΔYHB1 strain of* S. cerevisiae*. It has been reported that Ola1p suppresses antioxidant response via nontranscriptional mechanisms. Its upregulation in* E. coli* inhibits the ability of cells to scavenge damaging reactive oxygen species [51, 52]. All these mechanisms probably help* S. cerevisiae* to combat the deleterious effects on NO and survive normally.

In conclusion, flavohemoglobin deletion in* S. cerevisiae* affects translation of several proteins which have been reflected in label-free quantitative differential proteomic analysis. Although* S. cerevisiae* cells do not have a nitric oxide synthase orthologue in its genome sequence, still several reports indicated the presence of NO and RNS in it. It is conceivable that nitrosative stress would become a problem for* S. cerevisiae* in the absence of flavohemoglobin and global change at the translational level is an essential requirement for cell to cope with the stressed conditions. Further investigations are required to study the mechanisms of regulation of these pathways involving nitrosative stress.

## Supplementary Material

To better understand the physiological function of flavohemoglobin in yeast, in the present study we have carried out label free differential proteomics study using soluble extract of wild type (Y190) and flavohemoglobin deleted strains (ΔYHB1 mutant) of S. cerevisiae grown under fermentative conditions. Quantification was achieved by employing a label free system, whereby the summed intensity of the top three most intense peptides assigned to a protein is assumed to be proportional to the protein concentration, which can therefore be estimated by comparison with the three largest peptide intensities of an injected internal standard. Label free quantitation was performed using peak intensity measurements in Waters ExpressionE, which is part of PLGS 2.3. Supplementary Table 1 and Table 2 represent list of proteins identified in three replicates in Y190 and in ΔYHB1 respectively. List of proteins down regulated and up regulated due to YHB1 deletion are shown in Supplementary Table 3. Regulated proteins are classified using (DAVID) v6.7 functional annotation tool (Supplementary Table 4). Classified enriched Gene ontology (GO) terms were visualized using REViGO online and CYTOSCAPE v3.1.1 (Supplementary Table 5). Information of the regulated set of proteins that are used in STRING interaction network are shown are shown in (Supplementary Table 6).

## Figures and Tables

**Table 1:** List of downregulated proteins in ΔYHB1 cells of *S. cerevisiae*.

Gene ID	Protein description	log_2_⁡-fold change	*p* value
ILV5	Ketol-acid reductoisomerase, mitochondrial	−0.62	0.0002
BAT2	Branched-chain-amino acid aminotransferase, cytosolic	−1.05	0.0011
HSP26	Heat shock protein 26	−0.98	0.0015
HSP12	12 kDa heat shock protein	−0.75	0.0024
ARO2	Chorismate synthase	−1.11	0.0040
SHM2	Serine hydroxymethyltransferase, cytosolic	−0.89	0.0043
ASN1	Asparagine synthetase [glutamine-hydrolyzing] 1	−1.71	0.0045
ACO2	Probable aconitate hydratase 2	−0.78	0.0049
ARO8	Aromatic amino acid aminotransferase 1	−1.02	0.0050
ADE3	C-1-Tetrahydrofolate synthase, cytoplasmic	−0.54	0.0057
LYS20	Homocitrate synthase, cytosolic isozyme	−0.93	0.0079
ACO1	Aconitate hydratase, mitochondrial	−0.49	0.0099
HSP10	10 kDa heat shock protein, mitochondrial	−0.29	0.0104
DUG1	Cys-Gly metallopeptidase YFR044C	−0.22	0.0109
ENO1	Enolase 1	−0.73	0.0112
RPL34A	60S ribosomal protein L34-A	−0.70	0.0126
ARO4	Phospho-2-dehydro-3-deoxyheptonate aldolase, tyrosine-inhibited	−1.02	0.0137
HIS4	Histidine biosynthesis trifunctional protein	−2.82	0.0141
LYS1	Saccharopine dehydrogenase [NAD+, L-lysine-forming]	−1.33	0.0150
MET17	Protein MET17	−0.92	0.0164
ECM10	Heat shock protein SSC3, mitochondrial	−0.55	0.0174
LYS9	Saccharopine dehydrogenase [NADP+, L-glutamate-forming]	−1.17	0.0228
THR4	Threonine synthase	−0.22	0.0232
HSP82	ATP-dependent molecular chaperone HSP82	−1.02	0.0236
AAT2	Aspartate aminotransferase, cytoplasmic	−0.89	0.0246
BAT1	Branched-chain-amino acid aminotransferase, mitochondrial	−0.66	0.0249
DPS1	Aspartyl-tRNA synthetase, cytoplasmic	−0.59	0.0265
KGD1	2-Oxoglutarate dehydrogenase E1 component, mitochondrial	−0.38	0.0305
ASN2	Asparagine synthetase [glutamine-hydrolyzing] 2	−0.90	0.0321
SSB1	Heat shock protein SSB1	−2.02	0.0327
SOD1	Superoxide dismutase [Cu-Zn]	−0.24	0.0356
THS1	Threonyl-tRNA synthetase, cytoplasmic	−0.07	0.0362
ALD6	Magnesium-activated aldehyde dehydrogenase, cytosolic	−0.14	0.0364
LEU4	2-Isopropylmalate synthase	−1.35	0.0394
KRS1	Lysyl-tRNA synthetase, cytoplasmic	−0.72	0.0400
ILV3	Dihydroxy-acid dehydratase, mitochondrial	−0.98	0.0417
LEU1	3-Isopropylmalate dehydratase	−1.02	0.0451
AHP1	Peroxiredoxin type-2	−0.37	0.0460
RHR2	(DL)-Glycerol-3-phosphatase 1	−0.44	0.0503
ADH3	Alcohol dehydrogenase 3, mitochondrial	−0.58	0.0546

**Table 2:** List of upregulated proteins in ΔYHB1 cells of *S. cerevisiae*.

Gene ID	Protein description	log_2_⁡-fold change	*p* value
UGP1	UTP-glucose-1-phosphate uridylyltransferase	0.54	0.0003
TIF1	ATP-dependent RNA helicase eIF4A	1.08	0.0007
RPP1A	60S acidic ribosomal protein P1-alpha	1.32	0.0008
OLA1	Uncharacterized GTP-binding protein OLA1	1.03	0.0026
RPL6B	60S ribosomal protein L6-B	0.91	0.0032
YEF3	Elongation factor 3A	0.41	0.0043
RPL5	60S ribosomal protein L5	0.15	0.0056
ERG13	Hydroxymethylglutaryl-CoA synthase	0.37	0.0066
TEF1	Elongation factor 1-alpha	0.29	0.0084
RPS12	40S ribosomal protein S12	0.36	0.0085
YDJ1	Mitochondrial protein import protein MAS5	0.19	0.0090
RPS9B	40S ribosomal protein S9-B	0.09	0.0111
EFB1	Elongation factor 1-beta	0.30	0.0116
RPS7A	40S ribosomal protein S7-A	0.21	0.0119
CHC1	Clathrin heavy chain	0.70	0.0125
YDR365W-B	Transposon Ty1-LR4 Gag-Pol polyprotein	0.24	0.0131
CDC60	Leucyl-tRNA synthetase, cytoplasmic	0.30	0.0138
RPL20A	60S ribosomal protein L20-A	0.14	0.0148
SEC53	Phosphomannomutase	0.70	0.0152
FAS1	Fatty acid synthase subunit beta	0.37	0.0159
PSA1	Mannose-1-phosphate guanylyltransferase	0.63	0.0164
TEF4	Elongation factor 1-gamma 2	0.32	0.0173
ANB1	Eukaryotic translation initiation factor 5A-1	0.28	0.0173
CDC19	Pyruvate kinase 1	0.10	0.0183
HXK1	Hexokinase-1	0.34	0.0232
RPL16A	60S ribosomal protein L16-A	0.16	0.0259
RPL7A	60S ribosomal protein L7-A	0.12	0.0262
RPL9A	60S ribosomal protein L9-A	0.19	0.0271
TKL1	Transketolase 1	0.21	0.0280
HSC82	ATP-dependent molecular chaperone HSC82	0.55	0.0329
SAH1	Adenosylhomocysteinase	0.23	0.0336
SSZ1	Ribosome-associated complex subunit SSZ1	0.30	0.0345
TDH2	Glyceraldehyde-3-phosphate dehydrogenase 2	0.19	0.0346
GND1	6-Phosphogluconate dehydrogenase, decarboxylating 1	0.26	0.0352
RPS22A	40S ribosomal protein S22-A	0.19	0.0368
FAS2	Fatty acid synthase subunit alpha	0.31	0.0381
TDH3	Glyceraldehyde-3-phosphate dehydrogenase 3	0.12	0.0391
PDC1	Pyruvate decarboxylase isozyme 1	0.10	0.0407
RPL19A	60S ribosomal protein L19-A	0.06	0.0419
RPL2A	60S ribosomal protein L2-A	0.19	0.0468
RPL12A	60S ribosomal protein L12-A	0.23	0.0483

**Table 3:** Uniquely identified proteins in three replicate experiments in Y190 and ΔYHB1 cells of *S. cerevisiae*.

Gene ID	Description
Unique in Y190	
BNA1	3-Hydroxyanthranilic acid dioxygenase
ARG1	Argininosuccinate synthase
YHB1	Nitric oxide oxidoreductase; flavohemoglobin
IDH1	Subunit of mitochondrial NAD (+)-dependent isocitrate dehydrogenase
THR1	Homoserine kinase
PCK1	Phosphoenolpyruvate carboxykinase
ADE1	N-Succinyl-5-aminoimidazole-4-carboxamide ribotide synthetase
PYC1	Pyruvate carboxylase isoform, cytoplasmic
RNR2	Ribonucleoside-diphosphate reductase, small subunit

Unique in ΔYHB1	
CBF5	Pseudouridine synthase catalytic subunit of box H/ACA snoRNPs
PDB1	E1 beta subunit of the pyruvate dehydrogenase (PDH) complex
RPL37A	Ribosomal 60S subunit protein
RPL37B	Ribosomal 60S subunit protein

## References

[1] Hardison R. (1998). Hemoglobins from bacteria to man: evolution of different patterns of gene expression. *Journal of Experimental Biology*.

[2] Zhu H., Riggs A. F. (1992). Yeast flavohemoglobin is an ancient protein related to globins and a reductase family. *Proceedings of the National Academy of Sciences of the United States of America*.

[3] Poole R. K., Hughes M. N. (2000). New functions for the ancient globin family: bacterial responses to nitric oxide and nitrosative stress. *Molecular Microbiology*.

[4] Liu L., Zeng M., Hausladen A., Heitman J., Stamler J. S. (2000). Protection from nitrosative stress by yeast flavohemoglobin. *Proceedings of the National Academy of Sciences of the United States of America*.

[5] Membrillo-Hernández J., Coopamah M. D., Anjum M. F. (1999). The flavohemoglobin of *Escherichia coli* confers resistance to a nitrosating agent, a ‘nitric oxide releaser,’ and paraquat and is essential for transcriptional responses to oxidative stress. *The Journal of Biological Chemistry*.

[6] Gardner P. R., Gardner A. M., Martin L. A., Salzman A. L. (1998). Nitric oxide dioxygenase: an enzymic function for flavohemoglobin. *Proceedings of the National Academy of Sciences of the United States of America*.

[7] Hausladen A., Gow A. J., Stamler J. S. (1998). Nitrosative stress: metabolic pathway involving the flavohemoglobin. *Proceedings of the National Academy of Sciences of the United States of America*.

[8] Poole R. K., Anjum M. F., Membrillo-Hernández J., Kim S. O., Hughes M. N., Stewart V. (1996). Nitric oxide, nitrite, and Fnr regulation of hmp (flavohemoglobin) gene expression in *Escherichia coli* K-12. *Journal of Bacteriology*.

[9] Membrillo-Hernandez J., Coopamah M. D., Channa A., Hughes M. N., Poole R. K. (1998). A novel mechanism for upregulation of the *Escherichia coli* K-12 *hmp* (flavohaemoglobin) gene by the ‘NO releaser’, *S*-nitrosoglutathione: nitrosation of homocysteine and modulation of MetR binding to the *glyA-hmp* intergenic region. *Molecular Microbiology*.

[10] Crawford M. J., Goldberg D. E. (1998). Role for the *Salmonella* flavohemoglobin in protection from nitric oxide. *The Journal of Biological Chemistry*.

[11] Stevanin T. M., Poole R. K., Demoncheaux E. A. G., Read R. C. (2002). Flavohemoglobin Hmp protects *Salmonella enterica* serovar typhimurium from nitric oxide-related killing by human macrophages. *Infection and Immunity*.

[12] Bang I.-S., Liu L., Vazquez-Torres A., Crouch M.-L., Stamler J. S., Fang F. C. (2006). Maintenance of nitric oxide and redox homeostasis by the *Salmonella* flavohemoglobin Hmp. *The Journal of Biological Chemistry*.

[13] Keilin D., Tissieres A. (1954). Hemoglobin in certain strains of yeast *Saccharomyces cerevisiae*. *Biochemical Journal*.

[14] Lewinska A., Bartosz G. (2006). Yeast flavohemoglobin protects against nitrosative stress and controls ferric reductase activity. *Redox Report*.

[15] Hromatka B. S., Noble S. M., Johnson A. D. (2005). Transcriptional response of *Candida albicans* to nitric oxide and the role of the YHB1 gene in nitrosative stress and virulence. *Molecular Biology of the Cell*.

[16] Crawford M. J., Sherman D. R., Goldberg D. E. (1995). Regulation of *Saccharomyces cerevisiae* flavohemoglobin gene expression. *The Journal of Biological Chemistry*.

[17] Lacelle M., Kumano M., Kurita K., Yamane K., Zuber P., Nakano M. M. (1996). Oxygen-controlled regulation of the flavohemoglobin gene in *Bacillus subtilis*. *Journal of Bacteriology*.

[18] Cassanova N., O'Brien K. M., Stahl B. T., McClure T., Poyton R. O. (2005). Yeast flavohemoglobin, a nitric oxide oxidoreductase, is located in both the cytosol and the mitochondrial matrix: effects of respiration, anoxia, and the mitochondrial genome on its intracellular level and distribution. *The Journal of Biological Chemistry*.

[19] Zhao X.-J., Raitt D., Burke P. V., Clewell A. S., Kwast K. E., Poyton R. O. (1996). Function and expression of flavohemoglobin in *Saccharomyces cerevisiae*. Evidence for a role in the oxidative stress response. *Journal of Biological Chemistry*.

[20] Buisson N., Labbe-Bois R. (1998). Flavohemoglobin expression and function in *Saccharomyces cerevisiae*. No relationship with respiration and complex response to oxidative stress. *The Journal of Biological Chemistry*.

[21] Bhattacharjee A., Majumdar U., Maity D. (2010). Characterizing the effect of nitrosative stress in *Saccharomyces cerevisiae*. *Archives of Biochemistry and Biophysics*.

[22] Bhattacharjee A., Majumdar U., Maity D. (2009). In vivo protein tyrosine nitration in *S. cerevisiae*: identification of tyrosine-nitrated proteins in mitochondria. *Biochemical and Biophysical Research Communications*.

[23] Panja C., Ghosh S. (2014). Detection of in vivo protein tyrosine nitration in petite mutant of *Saccharomyces cerevisiae*: consequence of its formation and significance. *Biochemical and Biophysical Research Communications*.

[24] Sahoo R., Bhattacharjee A., Majumdar U., Ray S. S., Dutta T., Ghosh S. (2009). A novel role of catalase in detoxification of peroxynitrite in *S. cerevisiae*. *Biochemical and Biophysical Research Communications*.

[25] Bradford M. M. (1976). A rapid and sensitive method for the quantitation of microgram quantities of protein utilizing the principle of protein-dye binding. *Analytical Biochemistry*.

[26] Silva J. C., Gorenstein M. V., Li G.-Z., Vissers J. P. C., Geromanos S. J. (2006). Absolute quantification of proteins by LCMSE: a virtue of parallel MS acquisition. *Molecular and Cellular Proteomics*.

[27] Shen Z., Li P., Ni R.-J. (2009). Label-free quantitative proteomics analysis of etiolated maize seedling leaves during greening. *Molecular and Cellular Proteomics*.

[28] da Huang W., Sherman B. T., Lempicki R. A. (2009). Systematic and integrative analysis of large gene lists using DAVID bioinformatics resources. *Nature Protocols*.

[29] Huang D. W., Sherman B. T., Lempicki R. A. (2009). Bioinformatics enrichment tools: paths toward the comprehensive functional analysis of large gene lists. *Nucleic Acids Research*.

[30] Schlicker A., Domingues F. S., Rahnenführer J., Lengauer T. (2006). A new measure for functional similarity of gene products based on Gene Ontology. *BMC Bioinformatics*.

[31] Supek F., Bošnjak M., Škunca N., Šmuc T. (2011). Revigo summarizes and visualizes long lists of gene ontology terms. *PLoS ONE*.

[32] Franceschini A., Szklarczyk D., Frankild S. (2013). STRING v9.1: protein-protein interaction networks, with increased coverage and integration. *Nucleic Acids Research*.

[33] Hooper S. D., Bork P. (2005). Medusa: a simple tool for interaction graph analysis. *Bioinformatics*.

[34] Kim S. O., Orii Y., Lloyd D., Hughes M. N., Poole R. K. (1999). Anoxic function for the *Escherichia coli* flavohaemoglobin (Hmp): reversible binding of nitric oxide and reduction to nitrous oxide. *FEBS Letters*.

[35] Horan S., Bourges I., Meunier B. (2006). Transcriptional response to nitrosative stress in *Saccharomyces cerevisiae*. *Yeast*.

[36] Duan X., Yang J., Ren B., Tan G., Ding H. (2009). Reactivity of nitric oxide with the (4Fe-4S) cluster of dihydroxyacid dehydratase from *Escherichia coli*. *Biochemical Journal*.

[37] Bhattacharjee J. K. (1985). *α*-Aminoadipate pathway for the biosynthesis of lysine in lower eukaryotes. *Critical reviews in microbiology*.

[38] Zabriskie T. M., Jackson M. D. (2000). Lysine biosynthesis and metabolism in fungi. *Natural Product Reports*.

[39] Quezada H., Marín-Hernández A., Aguilar D. (2011). The Lys20 homocitrate synthase isoform exerts most of the flux control over the lysine synthesis pathway in *Saccharomyces cerevisiae*. *Molecular Microbiology*.

[40] Karsten W. E., Reyes Z. L., Bobyk K. D., Cook P. F., Chooback L. (2011). Mechanism of the aromatic aminotransferase encoded by the Aro8 gene from *Saccharomyces cerevisiae*. *Archives of Biochemistry and Biophysics*.

[41] Hyduke D. R., Jarboe L. R., Tran L. M., Chou K. J. Y., Liao J. C. (2007). Integrated network analysis identifies nitric oxide response networks and dihydroxyacid dehydratase as a crucial target in *Escherichia coli*. *Proceedings of the National Academy of Sciences of the United States of America*.

[42] Richardson A. R., Payne E. C., Younger N. (2011). Multiple targets of nitric oxide in the tricarboxylic acid cycle of *Salmonella enterica* serovar typhimurium. *Cell Host and Microbe*.

[43] Brown S. M., Upadhya R., Shoemaker J. D., Lodge J. K. (2010). Isocitrate dehydrogenase is important for nitrosative stress resistance in *Cryptococcus neoformans*, but oxidative stress resistance is not dependent on glucose-6-phosphate dehydrogenase. *Eukaryotic Cell*.

[44] Missall T. A., Pusateri M. E., Donlin M. J., Chambers K. T., Corbett J. A., Lodge J. K. (2006). Posttranslational, translational, and transcriptional responses to nitric oxide stress in *Cryptococcus neoformans*: implications for virulence. *Eukaryotic Cell*.

[45] Quezada H., Marín-Hernández A., Arreguín-Espinosa R., Rumjanek F. D., Moreno-Sánchez R., Saavedra E. (2013). The 2-oxoglutarate supply exerts significant control on the lysine synthesis flux in *Saccharomyces cerevisiae*. *FEBS Journal*.

[46] Parsyan A., Svitkin Y., Shahbazian D. (2011). MRNA helicases: the tacticians of translational control. *Nature Reviews Molecular Cell Biology*.

[47] Linder P., Slonimski P. P. (1989). An essential yeast protein, encoded by duplicated genes TIF1 and TIF2 and homologous to the mammalian translation initiation factor eIF-4A, can suppress a mitochondrial missense mutation. *Proceedings of the National Academy of Sciences of the United States of America*.

[48] Svitkin Y. V., Pause A., Haghighat A. (2001). The requirement for eukaryotic initiation factor 4A.elF4A; in translation is in direct proportion to the degree of mRNA 5 secondary structure. *RNA*.

[49] Sakasegawa Y., Hachiya N. S., Tsukita S., Kaneko K. (2003). Ecm10p localizes in yeast mitochondrial nucleoids and its overexpression induces extensive mitochondrial DNA aggregations. *Biochemical and Biophysical Research Communications*.

[50] Mao R.-F., Rubio V., Chen H., Bai L., Mansour O. C., Shi Z.-Z. (2013). OLA1 protects cells in heat shock by stabilizing HSP70. *Cell Death and Disease*.

[51] Zhang J., Rubio V., Lieberman M. W., Shi Z.-Z. (2009). OLA1, an Obg-like ATPase, suppresses antioxidant response via nontranscriptional mechanisms. *Proceedings of the National Academy of Sciences of the United States of America*.

[52] Wenk M., Ba Q., Erichsen V. (2012). A universally conserved ATPase regulates the oxidative stress response in *Escherichia coli*. *The Journal of Biological Chemistry*.

